# Sketch of 2018 dengue outbreak in a megacity, Bangladesh

**DOI:** 10.1186/s41182-022-00470-z

**Published:** 2022-10-26

**Authors:** Mohammad Robed Amin, Mohammad Rafiqul Islam, Muktadir Bhuiyan, Md. Shahnoor Islam, Fathema Islam, Habiba Jannatun Tuli, Afra Nawar, Tamanna Tabassum, Jannatul Fardous, Mohammad Jahid Hasan

**Affiliations:** 1 Department of Medicine, Dhaka Medical College, Dhaka, Bangladesh; 2 Department of Medicine, Saheed Suhrawardy Medical College, Dhaka, Bangladesh; 3 Pi Research Consultancy Center, Dhaka, Bangladesh; 4 Tropical and Infectious Disease Division, Tropical Disease and Health Research Center, Dhaka, Bangladesh

**Keywords:** Epidemiology, Dengue, Dengue fever, Epidemic, Outbreak

## Abstract

**Background:**

Dengue has become a major public health threat in Bangladesh since 2000, when the first outbreak was reported. Each outbreak has distinct characteristics, and thus, the report of the outbreak helps to understand the disease process and subsequent clinical management of these patients. On that ground, the study was designed to sketch the clinico-epidemiological characteristics of the 2018 dengue outbreak in Bangladesh.

**Methods:**

This hospital-based cross-sectional study was conducted in one of the largest public medical college hospitals and a single private hospital located in the southern and northern parts of the megacity of the country. A total of 297 confirmed dengue cases were assessed with a preformed pretested questionnaire. Clinico-epidemiological and laboratory parameters were reported along with sociodemographic details. Statistical analysis was performed with SPSS 20.

**Results:**

Male patients were predominantly affected by dengue infection. The mean age of the patients was 31.24 ± 13.99 (SD) years, with a range from 2 to 85 years. Eighty-two percent of patients reported from the Dhaka metropolitan city. The highest percentage of cases (37.1%) was isolated from Bansree, Dhaka city, followed by Rampura (21.4%) and Khilgaon (6.2%). In addition to common symptoms, e.g., fever (90.6%), headache (90.6%), chills (81.8%), anorexia and vomiting (76.4%), backache, and redness of the eyes were two prominent symptoms that affected more than two-thirds of the study population. On the other hand, less common symptoms, such as cough, abdominal pain, and respiratory distress, were present in 39.7%, 33.7%, and 15.5% of patients, respectively. Overall, 17.6% of patients were hypotensive during admission, with a mean systolic blood pressure of 107.65 ± 18.17 (SD) mmHg. Other prominent signs were dehydration (80.5%) and rash (33%).

**Conclusion:**

This outbreak was especially characterized by gastrointestinal symptoms, which were predominant along with other typical features.

## Background

Dengue fever, caused by Dengue virus (DENV), a member of the *Flavivirus* family, has emerged as one of the most common vector-borne diseases caused by *Aedes* mosquitoes. This virus is endemic to 128 countries, infecting 3.9 billion people each year [1]. There has been a 400% rise in dengue incidence over a period of 13 years since 2000, posing an increased risk of premature mortality, particularly in developing countries [2]. The warm and humid climate, dense population, unforeseen urbanization, lack of effective vector control, and faulty junk storage patterns permitting water clogging in residential areas and open spaces provide a suitable roaming and breeding ground for female *Aedes* mosquitoes [3]. Dhaka, the capital of Bangladesh, which is home to more than 17 million people, experienced the first major dengue outbreak in 2000, with 5551 dengue-infected cases and a fatality of 93 [4, 5]. An increase in the frequency and magnitude of dengue outbreaks was observed in 2017 and 2018, with 2769 and 10,148 reported cases, respectively. The largest outbreak of dengue was observed in Dhaka, Bangladesh, in 2019, with a ten times higher number of cases in that year [6, 7].

Dengue infections can be asymptomatic or may result in symptomatic dengue fever (DF) or end up as severe disease, presenting as bleeding-prone dengue hemorrhagic fever (DHF) or plasma leakage-related dengue shock syndrome (DSS) [8]. Recently, severe symptoms have been reported to have appeared much earlier than expected, while early signs were apparently absent from patients [9]. The literature review revealed that the presentation of dengue syndrome was typical, with high-grade fever with typical purpuric rash and breakbone body ache as common manifestations, while few pediatric cases presented with gastroenteritis.

Moreover, some recent research works have hinted that transmission might occur mutely by a process called transmission–clearance trade-off by which a pathogen chooses survival over high virulence [10]. This scenario also has another face. From a clinical point of view, there is a shift in the clinical picture from predominant pain- and fever-associated symptoms to more obvious respiratory and abdominal symptoms that are evident in day-to-day practice [9]. The involvement of multiple viral serotypes and coinfection with other vector-borne viral infections might affect the clinical presentations and magnitude of the severity of dengue. Therefore, understanding the cases in each outbreak is needed to develop clear management guidelines for managing dengue cases. Considering this fact, the study was designed to sketch the characteristics of the dengue patients witnessing the 2018 outbreak in Bangladesh.

## Methods

### Study design and study location

This cross-sectional study was conducted in one public medical college hospital and one private hospital located in the Dhaka Metropolitan city, also known as the capital of Bangladesh. Dhaka is one of the most crowded megacities in the world situated on the banks of the river Buriganga, with approximately 20 million people living over 453 km^2^ [11, 12].

The overall spatial density is 41,000/km^2^. Since independence, it has become the center point of all key administrative, economic and educational activities in the country. Consequently, a larger proportion of the Bangladeshi population lives here and in an average 400,000 people arriving each year for grabbing the economic opportunities or to educate their children or simply enjoy the higher living standards [11]. This large population stresses overall vector control management and may have an impact on frequent dengue outbreaks in megacities.

Patients presenting with a history of fever irrespective of age and sex were approached for inclusion in this study. Data collection was performed for the period of 6 months extending from the month of April to September 2018, when the upsurge of the cases was documented here. Patients attending Dhaka Medical College Hospital (DMCH) and Farazi Hospital, Badda, Dhaka, were chosen as study participants. Dhaka Medical College Hospital (DMCH) is one of the largest subsidized public hospitals located in the southern part of Dhaka city and is equipped with multidisciplinary care and played a crucial role during the 2018 dengue outbreak. These hospitals usually received referrals mostly from the whole Dhaka city and surrounding districts, and deals with patients mostly come from lower- to middle-income backgrounds. On the other hand, Farazi Hospital at Badda—a private multifacility hospital located in the northern part of Dhaka city has served a number of dengue cases in that outbreak, usually dealing with patients from higher income groups. These two centers were selected purposefully to blend the disparity of the economic class and to facilitate the research more efficiently.

### Study population and sample size calculation

The sample size for the present study was calculated from the following formula:

$n=\frac{{z}^{2}pq}{{d}^{2}}$, where *z* = 1.96 for the 95% confidence level, *p* = assumed 50% and *d* = allowable error 5% (0.05). The estimated sample size was 384. Assuming a 5% nonresponse rate, we approached a total of 404 patients and finally included 297 patients for analysis.

Any patient, regardless of age, who presented with a fever (40 °C/104 °F) and at least two of the typical dengue symptoms during the febrile phase (2–7 days) was considered for inclusion in this study. The typical symptoms are severe headache, peri-orbital pain, muscle and joint pain, nausea, vomiting, swollen glands and rash [13]. Following proper clinical evaluation through detailed history taking, thorough clinical examination and investigations, confirmed cases of dengue fever were included in this study. The diagnosis of dengue fever was performed according to ‘Dengue Guidelines for Diagnosis, Treatment, Prevention and Control’ by the World Health Organization [14]. A total of 297 confirmed dengue cases were finally included. Any coinfection of dengue with other viral, protozoal, or bacterial infections and patients or attendants who did not give consent were excluded from the study.

### Study procedure

A semistructured pretested questionnaire was used for data collection. A detailed history was taken, including their residence and travel, days of illness and symptoms. Immediate clinical examination was performed in every patient with special attention to fever, vital signs, urine output, rash, organomegaly and, when needed, organ functions. On the day of admission, 5 ml of blood was drawn from the antecubital vein to test the complete blood count (CBC) with hematocrit and alanine transaminase (ALT), aspartate transaminase (AST) and nonstructural protein 1 (NS1) antigen for dengue confirmation. Any patient who met the clinical inclusion criteria but could not receive the NS1 antigen was confirmed by serological testing for dengue (IgM and IgG) ELISA. Depending on the severity and organ involvement, other tests, such as chest X-ray posterior–anterior (PA) view, ultrasonography (USG) of the whole abdomen, serum creatinine and other biochemistry, such as serum calcium, serum electrolytes, and blood glucose, were performed. All tests of patients enrolled in DMCH were performed in the Department of Hematology and Biochemistry of DMCH, while all tests of patients enrolled in Farazi Hospital were performed in the Department of Hematology, Biochemistry and Radiology of Farazi Hospital. Although all patients were managed and followed up according to the standard guidelines, the inclusion of those data was beyond the scope of the study.

### Ethical approval

The study protocol was reviewed and approved by the Ethical Review Committee of DMCH, and the whole study was conducted in accordance with the current Declaration of Helsinki. Prior to enrollment, informed written consent was obtained from each eligible participant. Before obtaining consent, the aim and objectives of this study were briefly explained along with potential risks and benefits. If the participant had any kind of query, those were properly answered. The right to withdraw from this study was adequately briefed, and it was also made clear that withdrawing from this study would not jeopardize their treatment or result in any kind of penalty.

### Statistical analysis

Statistical analysis was performed using SPSS (statistical package for social science)- V 20. Descriptive statistics were used to express the data and interpretation. Before starting the analysis, the normality test was performed to check whether the data were distributed normally. In this study, quantitative continuous variables are expressed as the mean ± SD, while qualitative variables are expressed as the frequency and percentage.

## Results

The mean age was 31.24 ± 13.99 (SD) years, ranging from 2 to 85 years (Table 1). Overall, 98% of patients were enrolled from the adult medicine department, while 2% were only from pediatrics. Among the studied cases, males were predominant, with a ratio of 3:2 (60% vs 40%) (Table 1).

**Table 1:** Age and gender distribution of the dengue patients

	*n*	%*	Mean ± SD
Age group (years) (*N* = 291)			
≤ 10	7	2.4	31.24 ± 13.99
11–20	52	17.5	
21–30	125	42.1	
31–40	47	15.8	
41–50	34	11.4	
51–60	14	4.7	
61–70	8	2.7	
> 70	4	1.3	
Gender (*N* = 289)			
Male	175	60.6	
Female	114	39.4	

^*^Percentage was calculated based on the collected data

**Fig. 1:**
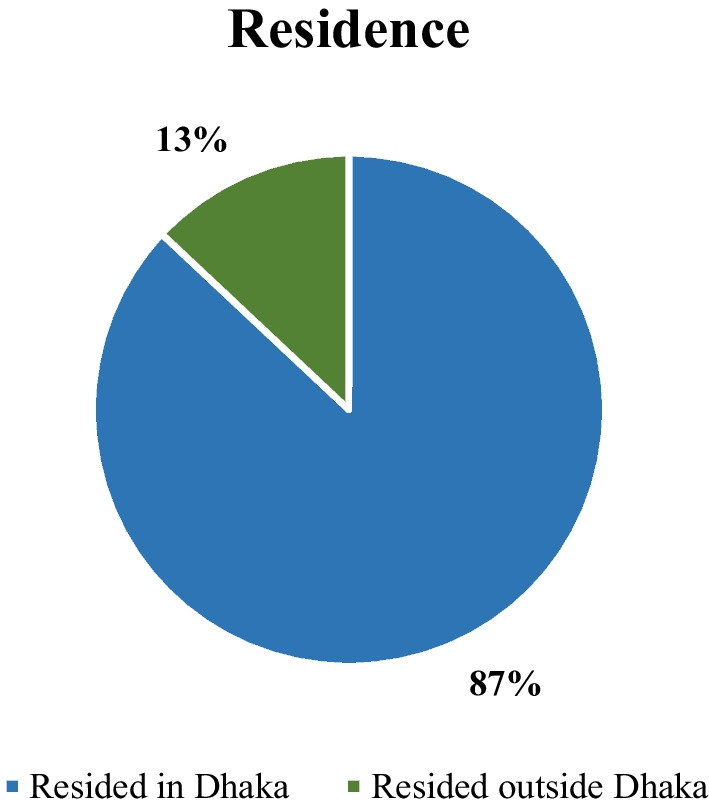
Residence-wise distribution of dengue patients

Approximately 87% of the total cases reside in this megacity, Dhaka. The location of the cases was confirmed from the history of the patients and is sketched in Fig. 1.

The clinical parameters of the dengue cases are summarized in Table 2. The common symptoms were fever, headache, chills, anorexia, vomiting, backache, neckache, retro-orbital pain, red eyes, cough and abdominal pain. Overall, gastrointestinal symptoms were predominant, as evidenced by a higher proportion of nausea and vomiting (76.4%), followed by diarrhea (38.7%), abdominal pain (33.7%), and bleeding per stool (6.1%) (Table 2). Clinical signs on admission day were predominantly dictated by dehydration, which was approximately 80% during admission. The mean pulse was 82.65 ± 11.49 b/min, while systolic blood pressure (SBP) was 107.65 ± 18.17 and diastolic blood pressure (DBP) was 72.35 ± 12.65 (SD) mmHg (Table 2). The mean hematocrit was 41.24% (SD 5.47). The mean white blood cell and platelet counts were 5818.20 and 123,991/mm^3^, respectively (Table 2). However, the pattern of fever was continuous in nature (83.5%) and subsided with taking paracetamol (87.5%) and NSAIDS (0.2%). The characteristics of rash were common in one-fourth of the patients, and almost half of the patients had no specific pattern (Table 3).

**Table 2:** Clinical parameters of dengue patients on admission

Clinical parameters	*n*	%
Symptoms (*n* = 297)*		
Fever	287	96.6
Severe headache	269	90.6
Retroorbital pain	151	50.8
Redness of eye	195	65.7
Chill	243	81.8
Back pain	218	73.4
Neck pain	120	40.4
Sore throat	87	29.3
Anorexia	239	80.5
Nausea and vomiting	227	76.4
Diarrhea (> 3 motions/day)	115	38.7
Rash	50	16.8
Blood cough	4	1.3
Joint pain	87	27.9
Body ache/myalgia	79	26.59
Hemoptysis	2	0.7
Vertigo	3	1.0
Earache	2	0.7
Abdominal pain	100	33.7
Jaundice	8	2.7
Cough	118	39.7
Respiratory distress	46	15.5
Pallor	14	4.7
Convulsion	4	1.3
Coma	6	2.0
Cool skin	10	3.4
Blood in stool	18	6.1
Nasal bleeding	4	1.3
Gum bleeding	6	2.0
Hematuria	4	1.3
Vaginal bleeding	11	3.7
Less urine output	23	7.7
Signs*		
Cyanosis (*n* = 291)	4	1.3
Anemia (*n* = 291)	4	1.3
Dehydration (*n* = 291)	239	82.1
Jaundice (*n* = 291)	4	1.3
Rash (*n* = 291)	98	33.6
Pleural effusion (*n* = 291)	10	3.4
Joint swelling (*n* = 291)	3	1.0
Palpable lymph node (*n* = 291)	4	1.3
Tourniquet test (*n* = 36)	6	16.7
Vital statistics**		
Pulse (/min) (*n* = 65)	82.65 ± 11.49	
SBP (mmHg) (*n* = 51)	107.65 ± 18.17	
DBP (mmHg) (*n* = 51)	72.35 ± 12.65	
Pulse pressure (mmHg) (*n* = 30)	27.50 ± 8.56	
Respiratory rate (/min) (*n* = 50)	17.46 ± 3.30	
Temperature (°F) (*n* = 32)	99.12 ± 0.79	

*SBP* systolic blood pressure, *DBP* diastolic blood pressure, *SD* standard deviation, *HCT* hematocrit, *WBC* white blood cell

Percentage was calculated based on the collected data

Variables were expressed as frequency, percentage* and mean ± standard deviation**

**Table 3:** Characteristics of fever and rash among dengue patients

	Frequency (*n*)	Percentage (%)
Pattern of fever (*n* = 256)		
Remittent	2	0.8
Intermittent	6	2.4
Continuous	248	96.8
Fever subside with taking (*n* = 262)		
Paracetamol	260	99.2
NSAID	2	0.8
Fever associated with (*n* = 192)		
Chills	141	73.5
Sweating	30	15.6
Shivering	21	10.9
Distribution of rash (*n* = 58)		
Localized	9	15.5
Universal	49	84.5
Specific site of rash (*n* = 58)		
Trunk	9	15.5
Upper limb	15	25.9
Flexor surface of lower limb	2	3.5
Extensor surface lower limb	2	3.5
Face	7	12.0
More than one site involvement	23	39.6
Type of rash (*n* = 58)		
Macular	6	10.3
Papular	15	25.8
Morbilliform	9	15.5
No specific pattern	28	48.4

NSAID: nonsteroidal anti-inflammatory drug; percentage was calculated based on the collected data

## Discussion

In 2018, the dengue outbreak was characterized by predominant gastrointestinal symptoms (more than 60%) in adult cases. In addition, a poor platelet count without shock was also obvious in a number of cases. In the last few years, dengue cases were determined by the triad of fever, pain and rash and managed by fluid replacement and cautious observation. In the most recent outbreak, along with other management, additional care was provided, particularly for patients presenting with gastrointestinal symptoms. Dengue syndrome has been endemic since its resurgence in Bangladesh in 2000 [15]. There has been improvement in the management and prevention point of view since then. Although dengue has been present every year with morbidity and few mortalities, a serious outbreak occurred in 2018 with a short time span. There have been more than 6000 cases with 28 deaths [16] reported officially, but as the reporting system is still not up to the mark, the numbers may be even more. Many patients with fewer symptoms or who were asymptomatic were not reported as a result of not visiting any health center. Many patients suffer without proper investigation, diagnosis and treatment due to a lack of facilities. Even in urban areas due to inhomogeneity of the care, confirmatory test RT-PCR was not available in most of the health care centers. A certain number of patients visit private chambers, and their investigations are also conducted in private facilities; those patients also remain underreported. Due to the lack of an effective community-level surveillance system, the overall incidence is still unknown. As a result, it was presumed that the total number of cases may be underreported compared with the original number. In response to the outbreak, the dengue case notification system was developed by the Directorate General Health System (DGHS), but only a few centers followed the steps. However, it can be hoped that using a modern tech-based surveillance system instead of a paper-based reporting system might improve the overall reporting system in the near future. Ensuring timely identification and quick reporting should be prioritized. In Malaysia, for surveillance of dengue, two distinct types of databases are used: one for registered cases and one for notified cases [18].

The 2018 outbreaks of dengue started with unusual rain during the month of April. The present study attempted to capture the clinical presentations of admitted dengue patients during this outbreak. In the 2018 outbreak, the present study showed that male sex (60.6%) and younger age groups (21–30 years) were predominant. Prior studies [16–19] also revealed similar findings related to age and morbidity. It is possible that frequent movement in these age groups due to the work environment led them to be susceptible to interacting with the *Aedes* mosquito. The availability of *Aedes* mosquitoes during the day and reluctance to use mosquito nets may be responsible for female housewives obtaining more dengue. Although this also applied for children, it was not similarly observed in the pediatric group, as there was less recruitment of pediatric cases because the designed private hospital predominantly addresses adult patients. However, overall, the cases observed around Dhaka city were predominantly adults rather than children. This indicates that there is a transition in the prevalence of dengue toward adults rather than children, which was observed for the first 10 years during the resurgence of dengue in Bangladesh. This has also been observed in Southeast Asian regions [20] as well as global scenarios [21].

The majority of patients were from North Dhaka, especially Banasree and Rampura. More than 50% of the cases were hailing from these two sites. This could be explained by overcrowding and substandard living amenities, which may be a suitable place for breeding vectors. The national program of vector control provides vector mapping based on vector surveillance. Based on the data, it was found that the density of vectors was uniformly present in the north and south of Dhaka Metropolitan city for 2014, 2016 and 2017, but these data were not representative of the whole country [22, 23]. Interestingly, in this study, 32 cases from different districts were treated in public hospitals who were referred from primary physicians or themselves. The involvement of different districts indicates the importance of surveillance and seroprevalence across the country rather than focusing on metropolitan cities only. The surveillance should cover not only the patient surveillance but also the vector surveillance and virus genomics.

The symptoms and signs were observed carefully in this study. Although the hallmark of dengue syndrome is still fever, the observation in this study revealed rash as an uncommon symptom (16.8%). The fever continued and was associated with body ache, neckache and anorexia, nausea and vomiting in the patients. The paradigm is gastrointestinal (GI) symptoms, including diarrhea, which were less clinically observed in previous years [16]. The presence of GI symptoms together with fever and chills are the reason for many dehydrated patients observed in this study (87%). Another paradigm was to see cough and respiratory distress frequently in this study (39.7% and 15.5%, respectively).

Bleeding manifestations at different sites (skin, gum, urine, GI tract and respiratory tract) were found sparsely, while jaundice, oliguria, convulsions and coma were rarely observed in this study, which indicates that expanded dengue syndrome is not a common phenomenon [16, 24]. There was tachycardia in only 9.8% of cases during presentation despite high fever, which indicates that relative bradycardia is a common finding in dengue, and physicians should keep this in mind during fever case evaluation. The systolic blood pressure and diastolic blood pressure were both approximately normal in most cases, but approximately 17.6% presented with hypotension, and 80% of cases presented with dehydration, indicating that the patients were not taking adequate fluid during the febrile phase. The appropriate fluids and adequate amounts in the febrile phase are also important, as the predominant GI symptoms make the patients more likely to become dehydrated. Only 3% of cases presented with pleural effusion, indicating delayed presentation, especially in public hospitals.

The study has a few limitations. First, the study site was selected purposely based on prior knowledge of the availability of a higher number of dengue cases. Viral serotype and other detailed investigations were not performed, mostly due to a lack of funding. However, the study findings will aid clinicians in understanding the characteristics of this outbreak and differences in the cases from other outbreaks. Eventually, the knowledge will assist in identifying the dengue case and prompt management. Additionally, we believe that the evidence will be helpful during revision of the national guideline for dengue management in the near future.

## Conclusion

The sketch of the 2018 outbreak witnessed in a megacity of Bangladesh revealed a preponderance of younger and male cases that were isolated from all over the city. Along with typical features, gastrointestinal symptoms were observed as a prominent feature of dengue in this outbreak.

## Data Availability

Data and material are available from the corresponding authors and could be shared upon reasonable request.

## Abbreviations

DENV: Dengue virus
DF: Dengue fever
DHF: Dengue hemorrhagic fever
DSS: Dengue shock syndrome
CBC: Complete blood count
ALT: Alanine transaminase
AST: Aspartate transaminase
NS1: Non-structural protein antigen
Ig M: Immunoglobulin M
IgG: Immunoglobulin G
ELISA: Enzyme linked immunosorbent assay
PA view: Posterior–anterior view
SPSS: Statistical Package for Social Science
GI: Gastrointestinal
